# Urine Cellular DNA Point Mutation and Methylation for Identifying Upper Tract Urinary Carcinoma

**DOI:** 10.3390/cancers14143537

**Published:** 2022-07-21

**Authors:** Wei Ouyang, Lufeng Luo, Junjie Zhang, Ran Xu, Qiang Lu, Zhenzhou Xu, Jianye Liu, Pei Li, Yaqun Zhang, Chuanchi Zhou, Wei Tang, Zhenting Wang, Manman Cao, Genming Xu, Long Wang

**Affiliations:** 1 Department of Urology, The Third Xiangya Hospital, Central South University, Changsha 410008, China; wei_ouyangtj@163.com (W.O.); liujianye810@163.com (J.L.); s1204705214@163.com (C.Z.); 2 Department of Urology, Xiangya Hospital, Central South University, Changsha 410013, China; 3 Hunan Key Laboratory of Molecular Precision Medicine, Xiangya Hospital, Central South University, Changsha 410013, China; 4 Department of Urology, The Second Xiangya Hospital, Central South University, Changsha 410028, China; xuran@csu.edu.cn; 5 Department of Urology, Hunan Provincial People’s Hospital, First Affiliated Hospital of Hunan Normal University, Changsha 410002, China; luqiang804@163.com; 6 Department of Urology, Hunan Cancer Hospital, The Affiliated Cancer Hospital of Xiangya School of Medical, Central South University, Changsha 410013, China; xuzhenzhou@163.com; 7 Yearth Biotechnology Co., Ltd., Changsha 410205, China; lipei@yearth.cn (P.L.); tangwei@yearth.cn (W.T.); caomm@yearth.cn (M.C.); 8 Department of Urology, Beijing Hospital, Beijing 100000, China; zhangyaqun3365@bjhmoh.cn; 9 National Center of Gerontology, National Health Commission, Beijing 100000, China; 10 Institute of Geriatric Medicine, Chinese Academy of Medical Sciences, Beijing 100000, China; 11 Affiliated Haikou Hospital of Xiangya Medical College, Central South University, Haikou 570100, China; zhentingwang@csu.edu.cn

**Keywords:** upper tract urinary carcinoma, liquid biopsy, DNA methylation, gene mutation

## Abstract

**Simple Summary:**

It’s difficult to detect upper tract urothelial carcinoma at early stage. Invasive testing may increase risk of cancer recurrences in the bladder after radical nephroureterectomy. Thus, in the present study, we incorporated two-gene mutation and methylation biomarkers to conduct the diagnostic tool of upper tract urothelial carcinoma and performed external validation to investigate the utility and stability of the optimal panel. It showed a highly specific and robust performance. It may be used as a replaceable approach for early detection of upper tract urothelial carcinoma, resulting in less extensive examinations in patients at low risk.

**Abstract:**

Background: To improve the selection of patients for ureteroscopy, avoid excessive testing and reduce costs, we aimed to develop and validate a diagnostic urine assay for upper tract urinary carcinoma (UTUC). Methods: In this cohort study we recruited 402 patients from six Hunan hospitals who underwent ureteroscopy for hematuria, including 95 patients with UTUC and 307 patients with non-UTUC findings. Midstream morning urine samples were collected before ureteroscopy and surgery. DNA was extracted and qPCR was used to analyze mutations in *TERT* and *FGFR3* and the methylation of *NRN1*. In the training set, the random forest algorithm was used to build an optimal panel. Lastly, the Beijing cohort (*n* = 76) was used to validate the panel. Results: The panel combining the methylation with mutation markers led to an AUC of 0.958 (95% CI: 0.933–0.975) with a sensitivity of 91.58% and a specificity of 94.79%. The panel presented a favorable diagnostic value for UTUC vs. other malignant tumors (AUC = 0.920) and UTUC vs. benign disease (AUC = 0.975). Furthermore, combining the panel with age revealed satisfactory results, with 93.68% sensitivity, 94.44% specificity, AUC = 0.970 and NPV = 98.6%. In the external validation process, the model showed an AUC of 0.971, a sensitivity of 95.83% and a specificity of 92.31, respectively. Conclusions: A novel diagnostic model for analyzing hematuria patients for the risk of UTUC was developed, which could lead to a reduction in the need for invasive examinations. Combining *NRN1* methylation and gene mutation (*FGFR3* and *TERT*) with age resulted in a validated accurate prediction model.

## 1. Introduction

Urothelial carcinoma (UC) is one of the most common tumors, and it can affect the lower and the upper tract (known as UTUC) [1]. In fact, UTUC only accounts for approximately 5%~10% of UC cases [2]. Generally, to arrive at a correct diagnosis, patients need to receive extensive examinations, such as imaging (computed tomography urography (CTU) or magnetic resonance urography (MRU)), cytology and diagnostic ureteroscopy, etc. Of the available imaging techniques, CTU has the highest accuracy, with 92% sensitivity and 95% specificity [3]. However, CT fails to detect epithelial “flat lesions” without a mass or thickening of the urothelium. As a result, there are still a few cases that cannot be accurately diagnosed. Ureteroscopic biopsy is considered the gold standard in the diagnosis and surveillance of UTUC, displaying high accuracy (more than 90%) and an extremely low false-negative rate for determining the tumor grade [4]. However, several studies have shown a higher recurrence rate after radical nephroureterectomy (RNU) in patients who underwent preoperative diagnostic ureteroscopic biopsy [5,6]. In a previous study [7], the accuracy of barbotage cytology for UTUC reached 91%. Overall, non-invasive diagnostic tools as of yet display low sensitivity, whereas ureteroscopic biopsy may lead to a poor prognosis. Therefore, the development of a more effective diagnostic method is imperative for UTUC.

UTUC is closely associated with the prevalence of genomic alterations. Analysis of the genomic features of UTUC provides information on the risk of intravesical recurrence [8]. With the development of next-generation sequencing, it is getting easier to obtain the genomes of various species and to identify novel biomarkers in urinary carcinoma. The mutations in the *TERT* gene promoter have been detected in most cases of UC [9,10,11,12]. Furthermore, some vital oncogene mutations of *FGFR3*, TP53, RAS, and MDM2 have shown a vital value for the diagnosis of UTUC, with 82.2% sensitivity and 100% specificity [13]. DNA methylation is one of the most common epigenetic alterations and it plays a critical role in the early stages of tumorigenesis [14]. Unlike gene mutations, DNA methylation shows tissue- and cancer-specificity, which can be used to identify early-stage cancers [15]. Recently, Xu and colleagues [12] demonstrated that the use of TERT promoter mutation and ONECUT2 methylation as epigenetic biomarkers showed superior performance in the detection of UTUC (sensitivity of 94.0%, specificity of 93.1%).

Our previous studies [16,17] demonstrated that cell-free single-molecule unique primer extension resequencing technology was a promising method for liquid biopsy. Furthermore, in a prospective cohort study [18], our results showed a gene-mutation panel for urine sediments which was a good diagnostic tool for BC. To develop a diagnostic tool for UTUC, two commonly occurring mutations (*TERT* and *FGFR3*) in UTUC were added together with NRN1 methylation in our test panel.

## 2. Methods

### 2.1. Patients and Samples

In this study, all suspected UTUC patients were specially selected from ChiCTR2000029980 (http://www.chictr.org.cn/, accessed on 18 February 2020). A total of 402 hematuria patients were enrolled from six centers (Xiangya Hospital, the Second Xiangya Hospital, the Third Xiangya Hospital, Hunan Provincial People’s Hospital, Hunan Cancer Hospital, Haikou Municipal People’s Hospital), including 95 patients diagnosed with malignant UTUC (UTUC+) and an additional 307 patients who were diagnosed with non-UTUC (UTUC−). All included patients received a comprehensive examination, including cytology, ureteroscopy, abdominal ultrasound, CTU and MRU. Correspondingly, all patients’ diagnoses were confirmed by histopathological findings.

In the validation set, a total of 103 hematuria patients were recruited from Beijing hospital, of whom 76 were included to assess the effectiveness of the diagnostic model.

### 2.2. Sample Collection and Mutated Gene Detection

The methods of sample collection and DNA extraction were described in our previous study [18]. A 50 mL first-void urine sample was collected from each of the included patients. Midstream morning urine samples were collected before ureteroscopy and surgery and stored at 4 °C. For the sequencing design of the mutated gene, one can also refer to previous study [18]. The protocol of the gene mutation qPCR reaction system is presented in Supplementary Table S1.

### 2.3. Methylation Analysis

The *NRN1* methylation level was detected via quantitative real-time polymerase chain reaction analysis (qPCR). qPCR was performed according to standard protocols. The primer sequences used for qPCR are shown in Supplementary Table S2. GAPDH was set as the internal reference. The protocols of the NRN1 methylation qPCR reaction system are presented in Supplementary Table S3, respectively. The reaction program is shown in Supplementary Table S4.

### 2.4. Statistical Analysis and Logistic Regression Model

All statistical analyses were performed using SPSS, v.24 (IBM, Armonk, NY, USA) and GraphPad Prism v.8 (La Jolla, CA, USA). The characteristics of the included patients were compared using a *t*-test for continuous variables, the Mann–Whitney U test for non-normally distributed variables and the Chi-squared test for categorical variables. Univariate and multivariate logistic regression analyses were conducted to evaluate the association between UTUC and variables. The area under the curve (AUC) was used to evaluate the performance of models both in the training and validation sets. Adobe Photoshop CS6 was used for image processing. The model was initiated in the R package ‘glmnet’ (R version 3.5.1). *p* < 0.05 was considered significant.

## 3. Results

### 3.1. The Baseline Characteristics of Included Patients

**Figure 1:**
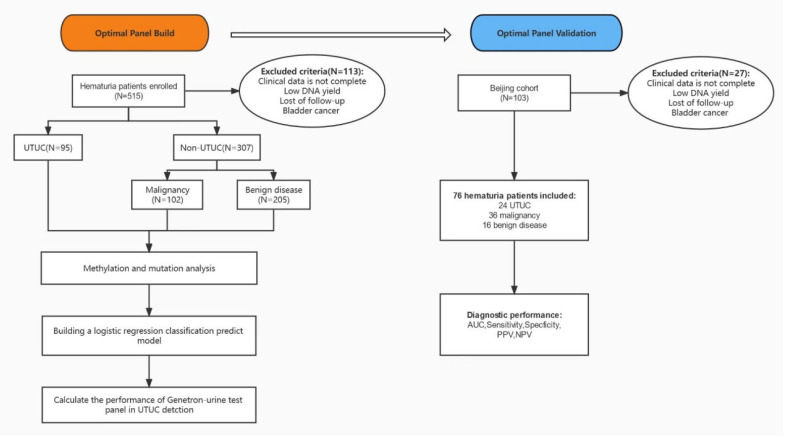
Sample and data processing workflow. The basic characteristics of all participants are shown in Table 1. In the UTUC+ group, 64 patients (67.36%) exhibited local disease, whereas 29 patients (30.53%) exhibited advanced disease. Furthermore, 17 patients (17.89%) had low-grade disease and 76 patients (80.00%) had high-grade disease. The differences in baseline characteristics (all *p* < 0.001) were significant between these two groups, aside from gender (*p* = 0.480). The details of mutated and methylated genes are shown in Figure 2.

In total, ninety-five UTUC+ patients and 307 UTUC− patients (102 other malignancies and 205 benign diseases) were included in this study (Figure 1).

### 3.2. Univariate Logistic Regression of Significant Features

The strength of each variable in assessing the UTUC risk was evaluated by means of univariate analysis, which is expressed using odds ratios (OR) (Supplementary Table S6). Mutated genes (*FGFR3* or *TERT*), *NRN1* methylation, and age showed significant impacts in evaluating UTUC risks (*p* < 0.001). For the panel, we integrated all these biomarkers to achieve superior performance in UTUC detection.

### 3.3. Multivariate Logistic Regression of Significant Features

The significant factors (age, gene mutation and NRN1 methylation) were combined to construct a multivariate logistic regression model according to the results of univariate logistic regression. The results are shown in Supplementary Table S7. Gene mutation (*p* = 0.002) and *NRN1* methylation (*p* < 0.001) played a vital role in diagnosing UTUC.

### 3.4. Gene Mutations and NRN1 Methylation Provided New Clinical Potential Applications

**Figure 3:**
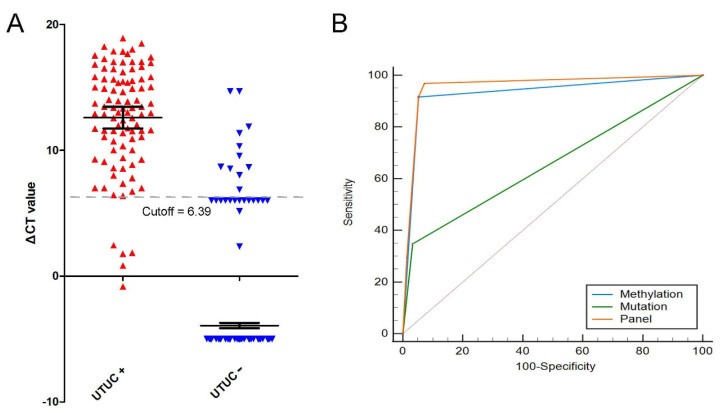
(**A**) The NRN1 methylation ΔCt-value distribution of the included patients. Red and blue triangles represent the NRN1 methylation level of individuals in UTUC and non-UTUC group, respectively. (**B**) ROC curve of NRN1 methylation (AUC = 0.932), gene mutation (AUC = 0.651) and the panel (AUC = 0.958), respectively.

An analysis was performed to confirm the best cutoff of *NRN1* methylation status. The *NRN1* methylation levels in all participants’ urine are shown in Figure 3A. With a cutoff of 6.39, the detection efficiency of *NRN1* methylation was the greatest, providing an AUC of 0.932 (Figure 3B), whereas the AUCs of gene mutation and of the entire panel were 0.657 and 0.958, respectively. Furthermore, the details of each diagnostic tool are shown in Table 2. Interestedly, the panel demonstrated superiority compared to gene mutation or *NRN1* methylation in isolation. Moreover, the panel was found to be a potential diagnostic tool for UTUC, displaying a sensitivity of 91.58%, a specificity of 94.79%, a positive predictive value (PPV) of 84.58% and a negative predictive value (NPV) of 97.36%.

### 3.5. Gene Mutations and NRN1 Methylation as a Diagnostic Tool to Identify UTUC and Benign Disease

**Figure 4:**
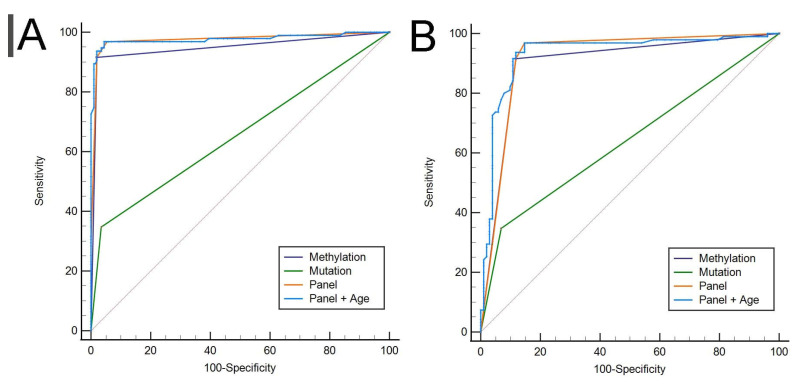
ROC curve of the of NRN1 methylation (AUC = 0.948), gene mutation (AUC = 0.657) and the panel (AUC = 0.975) in UTUC vs. begin disease (**A**) and of NRN1 methylation (AUC = 0.899), gene mutation (AUC = 0.639) and the panel (AUC = 0.920) in UTUC vs. other malignant tumors (**B**), respectively.

Patients with 205 benign diseases, including urolithiasis, urological infections, cysts and benign prostatic hyperplasia, were enrolled in this study. The ROC curves of the *NRN1* methylation (AUC = 0.948), gene mutation (AUC = 0.657) and panel (AUC = 0.975) results are shown in Figure 4A. The pairwise comparison of ROC curves demonstrated the same results as those obtained in the results for UTUC vs. other malignant tumors.

### 3.6. Gene Mutations and NRN1 Methylation as a Diagnostic Tool to Identify UTUC and Other Malignant Tumors

Patients with 102 other malignant tumors, including prostate cancer, renal cell carcinoma and penile cancer, were enrolled in this study. The ROC curves of *NRN1* methylation (AUC = 0.899), gene mutation (AUC = 0.639) and the panel (AUC = 0.920) are shown in Figure 4B. In the pairwise comparison of ROC curves, the panel provided superiority for UTUC detection, compared to *NRN1* (*p* = 0.029) and gene mutation (*p* < 0.001).

### 3.7. Panel Optimization

**Figure 5:**
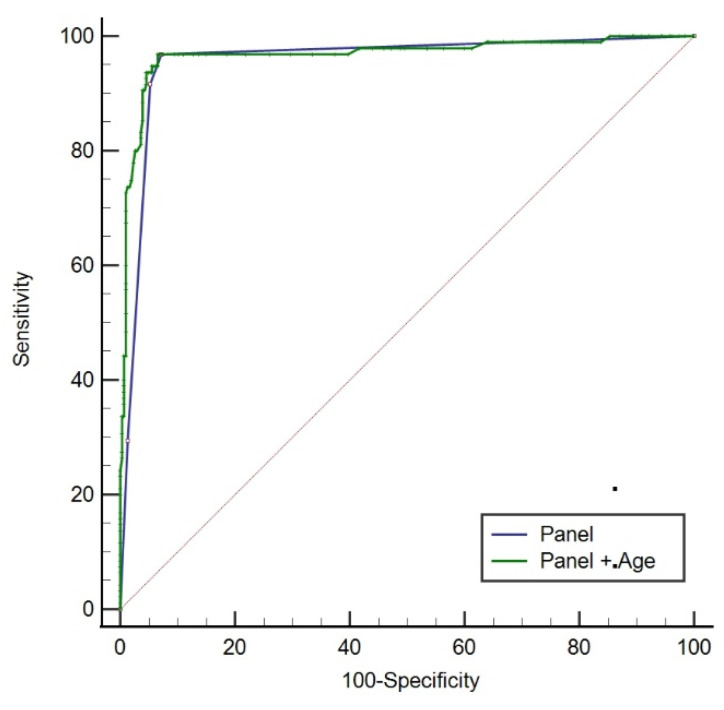
ROC curve of the panel (AUC = 0.958) and the panel combined with age (AUC = 0.968).

The results of univariate logistic regression showed that age was associated with pathology. Therefore, we analyzed the ROC curve (see Figure 5) for the model constructed with age and the panel, it had the largest AUC of 0.968 (*p* < 0.001), whereas the AUC of the panel was 0.958. The model with age, gene mutation and NRN1 methylation showed the optimal performance, with 93.68% sensitivity, 94.44% specificity, 86.46% PPV and 98.05% NPV (see Table 3). This model was considered optimal because it displayed the maximum sensitivity and specificity.

### 3.8. Novel Diagnostic Model, Cytology and FISH Comparison

We compared the diagnostic performances of the novel model, cytology and FISH in relation to UTUC (see Table 4). The sensitivity values of the novel model, cytology, and FISH in UTUC were 89/95 (93.68%), 39/95 (41.05%) and 81/95 (85.26%), respectively. This indicates that the performance of the novel model was superior to that of urine cytology and FISH. On the other hand, the specificity values of the novel model, FISH and urine cytology in UTUC were 290/307 (94.44%), 281/307 (91.53%) and 265/307 (86.32%), respectively. This means that both the novel model and FISH were superior to cytology. Overall, the novel model displayed significantly superior performance compared to conventional urine cytology and FISH in the diagnosis of UTUC. 

### 3.9. The Performance of the Detector in an Independent Validation Data Set

**Figure 6:**
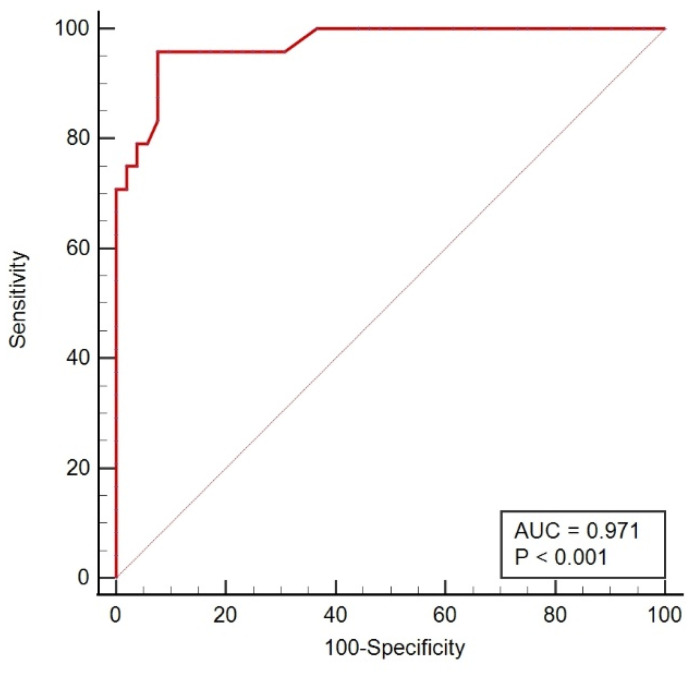
ROC curves of the optimal model in the validation set.

Seventy-six participants were recruited from Beijing hospital for external validation to test the reliability of the novel model. The optimal model showed excellent effectiveness, with an AUC of 0.971 in the validation set, a sensitivity of 95.83% and a specificity of 92.31% (see Figure 6).

## 4. Discussion

The continued development of new technologies is expected to improve the diagnosis and management of UTUC. Preoperative detection tools are essential to guide treatment decisions. Some epigenetic polymorphisms are strongly associated with increased cancer risk, which may lead to differences in individual susceptibility to these risk factors. Some risk factors and molecular pathways may play a vital role in both UTUC and BC [8]. Patients of Asian ethnicity appear to have more advanced and higher-grade disease compared to others at the time of initial disease diagnosis [19]. The early detection of UTUC is still a challenge; thus, it is necessary to develop more reliable detection tools [20,21,22]. Generally, the number and frequency of mutations in different biological samples may vary depending on the grade and stage of the cancer. Despite the limited number of cases, the results of this study showed that patients with malignant BC had the highest total number of mutations in their urine, whereas normal urine samples had the lowest average total number of mutations [18]. Since normal urinary epithelial cells lack TERT promoter and FGFR3 mutations, they may be ideal urinary biomarkers for identifying UTUC [23]. Indeed, the TERT promoter mutation has been previously used as a urine biomarker for identifying UC, presenting promising results [21,22,24,25,26]. Interestingly, Hosen et al. found that TERT promoter mutations may be present in urine as early as 10 years before the diagnosis of BC [25]. Both UTUC and BC originate from the uroepithelium, and are classified as transitional cell carcinomas; thus, this genetic alteration may also occur in the early stages of UTUC. The mutation frequency of TERT promoters in UTUC tumors was 28%, which was lower than that (44.3%) in UBCs [27]. *FGFR3* alterations occurred significantly more frequently in UTUC than in BC (40% vs. 20%) [28]. Furthermore, a study by Yujiro and colleagues [23] demonstrated that the specificity was 96.0% and the sensitivity was 78.6% in the diagnostic model combining TERT promoter, FGFR3 mutation and cytology results. Neuritin 1 (NRN1), also known as cpg15-1, is a GPI-anchored protein that is primarily involved in neuronal plasticity. Recently, several studies have shown that aberrant methylation in the promoter region of the nrn1 gene is associated with the development of tumors (e.g., gastric cancer and melanoma) [29,30]. In our study, we have identified for the first time that the methylation status of the NRN1 gene promoter in urine is a potential biomarker for detecting UTUC. However, the biological functions and mechanisms of NRN1 methylation have still not been fully elucidated and need to be further explored.

Interestingly, in our panel consisting of gene mutation and methylation, the model demonstrated superior performance in identifying UTUC, with a sensitivity of 96% and a specificity of 98%. In the past 5 years, nine articles on the genomics of UTUC have been published, using high-throughput sequencing (NGS) technology [8,31,32,33,34,35,36,37,38].

In our previous studies [16,17] we demonstrated that urine-based liquid biopsy is an effective detection tool for UC. Notably, the accuracy of the five-gene panel for the urine supernatant (AUC = 0.94) was superior to the seven genes for urine sediments (AUC = 0.91). Additionally, the highest cumulative mutation frequencies were found in *FGFR3* and *TERT*. In a single-gene diagnostic model, *TERT* and *FGFR3* showed superiority compared to the other genes. Hence, in this study we aimed to assess the diagnostic value of the model consisting of gene (*TERT* and *FGFR3*) mutation and *NRN1* methylation analyses.

Compared with UTUC−, the frequency of gene mutations in free DNA obtained from the urine supernatant of UTUC+ was significantly increased. Springer et al. [39] reported that mutant genes in urinary samples of UTUC patients in Taiwan could be detected at rates of 10.7% (*FGFR3* S249C), 7.1% (*TERT* C250T) and 25.0% (*TERT* C228T), whereas Hayashi and colleges [23] detected mutations at rates of 16.1% (*FGFR3* S249C), 7.1% (*TERT* C250T) and 39.3% (*TERT* C228T) in urinary samples in Japanese UTUC patients. The difference in detection rates may be due to differences in the cohorts investigated and in their exposure to aristolochic acid, arsenic and smoking [40,41,42]. In the present study, a new diagnostic model was used for distinguishing UTUC patients from hematuria patients and it showed excellent diagnostic value. Surprisingly, we observed the superiority of NRN1 methylation in UTUC diagnosis, and we found that it has high potential for clinical application. The use of the status of *NRN1* methylation to diagnose UTUC presents high sensitivity and specificity. The panel combining *TERT* and *FGFR3* mutation and *NRN1* methylation showed more superiority than those using single genes alone.

Furthermore, the NPV was 98.05% for the overall sample, whereas the PPV was 88.3% in the diagnostic model. Interestingly, two UTUC+ patients’ urinary CT scans showed no obvious abnormalities, whereas the panel presented a positive prediction. Subsequently, urothelial thickening was proven via ureteroscopy. Although CT has highly diagnostic accuracy for the detection of UTUC, epithelial “flat lesions” without a mass effect or urothelial thickening are generally not visible with CT. At this moment, the panel combining mutation with methylation could play a vital role in UTUC detection. Importantly, this is a noninvasive technology. Notably, a combined model with an NPV of 98.05%, in which the urine biopsy is implemented as a diagnostic tool, could lead to a reduction in the use of ureteroscopy. Thus, it may reduce costs and the risk of poor prognosis.

Due to the observational nature and the low incidence of UTUC, the composition of our sample was not comparable with the real-world data, and selection bias may have occurred. Secondly, mutant and methylated biomarkers are not only specific for UTUC. In some locally advanced prostate and renal carcinomas, cancer cells may be shed into the urine and make it difficult to identify UTUC using this model. Thirdly, some patients were excluded from our study because of low DNA yields. Additionally, the clinical application of the diagnostic tool may be hampered by unsuccessful DNA extraction.

## 5. Conclusions

We discovered that the *NRN1* methylation level showed superior performance in the detection of UTUC. Importantly, we developed an accurate testing panel combining significant gene mutation (*TERT* FGFR3) and *NRN1* methylation, and further optimized the diagnostic model by combining these factors with age. This panel provides high accuracy in UTUC detection, and could reduce costs and discomfort among patients.

## Figures and Tables

**Figure 2:**
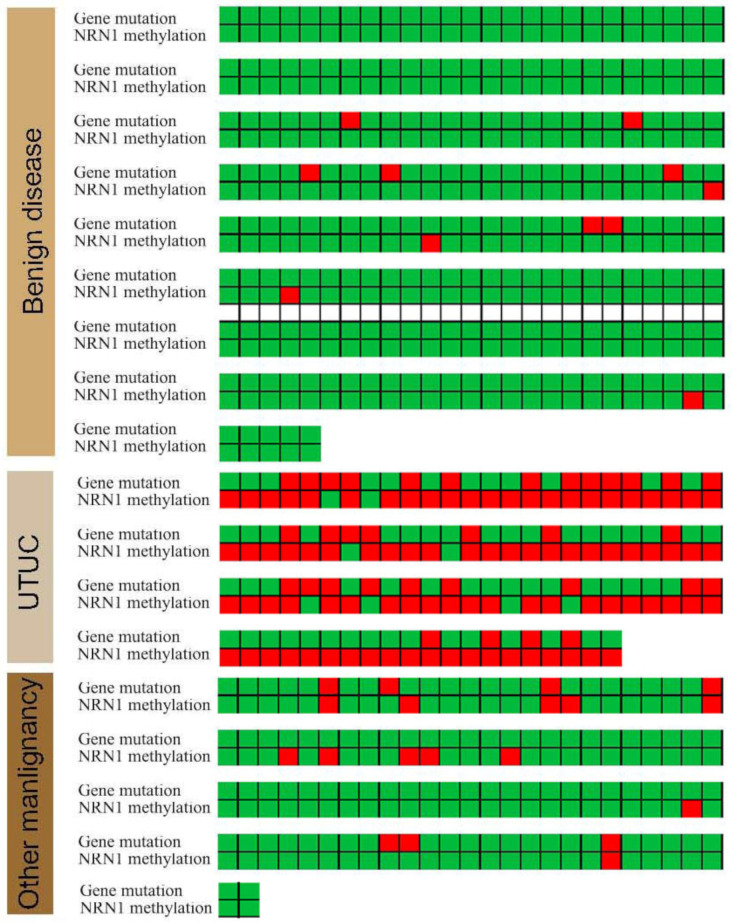
A heatmap showing details of mutated and methylated genes of the enrolled cases. Thirty-three (34.74%) UTUC patients exhibited mutated *TERT* or *FGFR3* genes, whereas 3 (1.46%) in the benign disease group and 7 (6.86%) in the other malignant tumor group exhibited these mutated genes. Moreover, the rate of *NRN1* methylation in the UTUC, benign disease and other malignant tumor groups were 91.58%, 1.95% and 11.76%, respectively. In the stage- and grade-specific analyses, methylation (*NRN1*) and mutation (*FGFR3* or *TERT*) were comparable in the Ta-2 vs. T3-4 stage and low-grade vs. high-grade analyses (Supplementary Table S5).

**Table 1:** Clinical and histopathological characteristics of enrolled cases.

Characteristic	Number of UTUC Patients (*n* = 95)	Number of Non-UTUC Patients (*n* = 307)	*p* Value
Age, *n* (%)	66.53 ± 11.14	58.93 ± 12.43	<0.001
Gender, *n* (%)			0.480
Male	62 (65.26)	188 (61.24)	
Female	33 (34.74)	119 (38.76)	
NRN1, *n* (%)			<0.001
Positive	87 (91.58)	16 (5.21)	
Negative	8 (8.42)	291 (94.79)	
Gene Mutation, *n* (%)			<0.001
Yes	33 (34.74)	10 (3.26)	
No	62 (65.26)	297 (96.74)	
Grade, *n* (%)		NA	NA
Low grade	17 (17.89)		
High grade	76 (80.00)		
Gx	2 (2.11)		
Stage		NA	NA
Ta-2	64 (67.36)		
T3,4	29 (30.53)		
Tx	2 (2.11)		

**Table 2:** The detection performance of three diagnostic tools in identifying UTUC.

Variables	Test Performance				
	AUC	Sensitivity (%)	Specificity (%)	PPV (%)	NPV (%)
Gene mutations	0.657	34.74	96.74	76.73	82.74
NRN1 methylation	0.932	91.58	94.79	84.58	97.36
Panel	0.958	91.58	94.79	84.58	97.36

**Table 3:** The detection performances of different models.

Variables of Models	Models with Different Features	
	Panel	Panel + Age
AUC	0.958 (0.933–0.975)	0.968 (0.945–0.983)
Sensitivity (%)	91.58 (84.15–96.37)	93.68 (86.83–97.65)
Specificity (%)	94.79 (91.70–97.02)	94.44 (92.55–97.57)
PPV (%)	84.58 (77.14–89.88)	86.46 (79.28–91.41)
NPV (%)	97.36 (94.92–98.67)	98.05 (95.73–99.11)

**Table 4:** Effectiveness of the novel model, cytology and FISH in the diagnosis of UTUC.

	Urinary Cell-Free DNA		FISH		Cytology	
	+	−	+	−	+	−
UTUC+	89	6	81	14	39	56
UTUC−	17	290	26	281	42	265
Sensitivity (%)	93.68		85.26		41.05	
Specificity (%)	94.44		91.53		86.32	

## Data Availability

The data used to support the findings of this study are available from the corresponding author upon request.

## Supplementary Materials

The following supporting information can be downloaded at: https://www.mdpi.com/article/10.3390/cancers14143537/s1. Supplementary Table S1. The protocol of FGFR3 and TERT mutation PCR reaction system. Supplementary Table S2. Primers for real-time quantitative PCR analysis. Supplementary Table S3. The protocol of NRN1 methylation PCR reaction system. Supplementary Table S4. The program of qPCR reaction. Supplementary Table S5. Results of stage and grade specific analyses. Supplementary Table S6. Univariable logistic regression analysis. Supplementary Table S7. Multivariate logistic regression analysis.

## Abbreviations

UBC: urothelial bladder carcinoma; UC: urothelial carcinoma; CT: computed tomography; UTUC: upper tract urothelial carcinoma; UC: urothelial carcinoma; AUC: area under the curve; PPV: positive predictive value; NPV: negative predictive value; CTU: computed tomography urography.
