# Supplementary material for: Urine Cellular DNA Point Mutation and Methylation for Identifying Upper Tract Urinary Carcinoma
Source: Cancers (Basel). 2022 Jul 21;14(14):3537. doi: 10.3390/cancers14143537 (PMC9319988; doi:10.3390/cancers14143537)
Supplement: Supplementary file 1 [file cancers-14-03537-s001.zip › cancers-1768678-supplementary.pdf]

Supplementary Table S1. The protocol of *FGFR3* and *TERT* mutation PCR reaction system.

DNA	50 ng
T228-F（20uM）	1 μL
T228-R（20uM）	1 μL
T228-FAM-MGB（10uM）	1 μL
F746-F（20uM）	1 μL
F746-R（20uM）	1 μL
F746-FAM-MGB（10uM）	1 μL
GAPDH-F（10uM）	0.5 μL
GAPDH-R（10uM）	0.5 μL
GAPDH-VIC-MGB（10uM）	0.5 μL
50×ROX Reference Dye	1 μL
2×AmpliTaq Gold™ 360 PCR Master Mix	25 μL
NF-H_2O	Add to 50μL

Supplementary Table S2. Primers for real-time quantitative PCR analysis.

**Primer name**	**Primer sequence (5′-3′)**
NRN1-F	AGATGTGTATAAGAGACAGTTGTAGGAAGAGCGAGGC
NRN1-R	AGATGTGTATAAGAGACAGTTACGTATCTACCCCAACG

Supplementary Table S3. The protocol of *NRN1* methylation PCR reaction system.

DNA	100 ng
676-bis-F4（20uM）	2 μL
676-bis-R3（20uM）	2 μL
676- bis-FAM 2-MGB（10uM）	1 μL
ACTB-bis-F2（10uM）	0.5 μL
ACTBis-R3（10uM）	0.5 μL
ACTB-bis-VIC-MGB（10uM）	0.5 μL
50×ROX Reference Dye	1 μL
2×AmpliTaq Gold™ 360 PCR Master Mix	25 μL
NF-H_2O	Add to 50μL

Supplementary Table S4. The program of qPCR reaction.

Number	Step	Temperature	Time	Cycles
1	Pre-denaturation	95℃	10min	1
2.1	Denaturation	95℃	15s	
2.2	Annealing	59℃	45s	15
2.3	Extension	72℃	1min	
3.1	Denaturation	95℃	15s	
3.2	Annealing	55℃	45s	
3.3	Extension，Fluorescence signal (FAM/VIC) acquisition	72℃	1min	35
4	Instrument cooling	40℃	5min	1

Supplementary Table S5. Results of stage and grade specific analyses.

	Ta-2	T3-4	P value	Low-grade	High-grade	P value
**Methylation**						
negative	7	1	0.428	1	6	1.000
positive	57	28		17	69	
**Mutation**						
negative	42	20	0.752	11	51	0.629
positive	22	9		7	25	

Supplementary Table S6. Univariable logistic regression analysis.

Variables	OR	95% CI	P value
**Gender**<br>male	1.443	0.81-2.56	0.211
**Age**	1.06	1.03-1.08	**<0.001**
**Mutations**<br>≥1 gene mutated	15.87	7.41-33.33	**<0.001**
**Methylation**<br>NRN1(△Ct<6.39)	200.00	83.33-500.00	**<0.001**
**Panel**	825.19	262.90-2590.10	**<0.001**

OR: odds ratio

Supplementary Table S7. Multivariate logistic regression analysis.

Variables	OR	95% CI	P value
**Age**	1.01	0.97-1.05	0.539
**Mutations**<br>≥1 gene mutated	9.35	2.35-37.04	**0.002**
**Methylation**<br>NRN1(△Ct<6.39)	166.67	62.50-500.00	**<0.001**

OR: odds ratio
